# The fusion of artificial intelligence and omics: a perspective toward 2035

**DOI:** 10.3332/ecancer.2026.2152

**Published:** 2026-06-18

**Authors:** Enrique Díaz Cantón

**Affiliations:** Oncology and Artificial Intelligence in Medicine, Instituto Universitario CEMIC, Buenos Aires C1431FWO, Argentina

**Keywords:** artificial intelligence, protein dynamics, world models, JEPA, AlphaFold, oncology, intrinsically disordered proteins, drug design, conformational dynamics, computational biology

## Abstract

Artificial intelligence (AI) has catalyzed a revolution in the life sciences, culminating in AlphaFold’s capacity to predict the static structure of proteins with atomic precision. However, this static view—analogous to a molecular radiograph—omits the fundamental dimension of protein dynamics, a critical factor for biological function and drug interaction. The phenomenon of induced fit, whereby proteins reconfigure upon ligand binding, remains largely beyond the reach of current models. This article posits that the next frontier in computational biology lies in the adoption of World Models—AI architectures such as Google DeepMind’s Genie 3 and Meta AI’s Joint-Embedding Predictive Architecture. Rather than predicting individual states, these models learn the underlying rules governing system evolution. We argue that applying these principles to molecular dynamics may enable the simulation of conformational trajectories, predicting how proteins move, flex, and respond to ligand binding. We explore the transformative implications for oncology, particularly in the design of allosteric modulators and in targeting historically “undruggable” proteins such as intrinsically disordered proteins. Finally, we delineate a roadmap toward 2035, highlighting challenges and opportunities at the convergence of generative AI and dynamic structural biology.

## Introduction

Molecular biology has entered a new era driven by artificial intelligence (AI). The development of AlphaFold by Google DeepMind, and its subsequent evolution, has largely solved the “protein folding problem”—one of biology’s most intractable questions for half a century [1, 2]. The capacity to predict the three-dimensional structure of virtually any protein from its amino acid sequence has provided the scientific community with an unprecedented structural universe, accelerating research across all fields of life sciences, from fundamental biology to drug design.

AlphaFold’s success has been followed by an explosion of specialized AI models addressing complementary facets of the central dogma. AlphaMissense, for instance, leverages structural predictions to classify the pathogenicity of millions of genetic variants, [3] while AlphaProteo ventures into de novo protein design, creating binders with unprecedented affinities and specificities [4]. More recently, AlphaGenome has extended this predictive power beyond proteins to the vast non-coding territory of the genome, predicting the effects of regulatory variants on gene expression and chromatin structure [5].

Nevertheless, despite these monumental advances, the AlphaFold suite and its derivatives share a fundamental conceptual limitation: they operate on a static representation of the molecular world. They provide us with a high-resolution snapshot—a “radiograph” of the protein in its lowest-energy state—but proteins are not rigid entities. They are dynamic molecular machines existing as an ensemble of conformational states in equilibrium, constantly fluctuating and moving to carry out their functions. This dynamics constitutes the heart of biological function, and its omission represents the principal bottleneck for the next generation of discoveries, especially in the development of precision oncology therapeutics.

### The problem: beyond static structure

The most significant limitation of current structural prediction models, including the advanced AlphaFold 3, is their inability to capture the inherent dynamics of biomolecules (Table 1) [2]. AlphaFold 3, while representing a qualitative leap in predicting the structure of multimolecular complexes (protein-ligand, protein-DNA), still generates a snapshot of the bound state. It does not predict the transition toward that state.

This problem manifests critically in the phenomenon of induced fit, a pillar of modern enzymology and pharmacology. When a drug or endogenous ligand approaches its target protein, it does not couple to a rigid active site like a key in a lock. Instead, the protein and ligand mutually recognize each other and, through a series of conformational rearrangements, the protein changes shape to embrace the ligand, optimizing interactions and stabilizing the complex. This “molecular embrace” is often what triggers cellular signaling or enzymatic inhibition.

AlphaFold gives us a high-quality image of the hand closed around the ball, but it does not show us the movie of how the hand closes. This omission has profound implications for precision oncology:

**“Undruggable” therapeutic targets.** Many of the most sought-after proteins in the fight against cancer—KRAS, c-Myc, p53—have long been considered “undruggable” precisely due to the absence of well-defined binding cavities in their apo (unbound) state. Their functional sites are often shallow or form transiently. Drug design for these targets depends not on finding a molecule that fits into an existing structure, but on designing one that can induce the formation of a new cavity and stabilize an inactive conformation.

**Allosteric modulation.** Allostery is the process whereby binding of an effector at one site on a protein (allosteric site) triggers a conformational change that modulates activity at a distant functional site. This is an immensely powerful therapeutic strategy, as it allows finer and more specific regulation of protein function. However, designing allosteric modulators is nearly impossible without understanding the dynamic communication pathways through the protein structure—something static models cannot provide.

**Intrinsically disordered proteins (IDPs).** A significant subset of cancer-implicated proteins, such as c-Myc and the tumor suppressor p53, lack a stable three-dimensional structure entirely or in large regions. They exist as a rapidly changing ensemble of conformations. AlphaFold, trained to predict a single low-energy structure, often “hallucinates” or imposes an ordered structure on these regions, failing to capture their essential dynamic nature [2].

To overcome these barriers, we need not better “radiographs” but “movies.” We need models that not only predict shape but simulate behavior.

### The proposed solution: world models for molecular dynamics (MD)

If the limitation is statics, the solution is dynamics. We propose that the next paradigm shift in computational biology will emerge from the adaptation of a new class of AI architectures known as World Models. A world model, unlike a purely predictive model, is not limited to mapping an input to an output. Its objective is to construct an internal, compressed representation of the principles governing an environment, enabling it to simulate or “dream” how that environment will evolve over time and in response to different actions [6].

The analogy is the difference between memorizing every possible move on a chessboard and understanding the underlying strategy of the game. Current models are masters at memorizing the “positions” of pieces (structures), but a world model learns the “rules” of movement (physics and thermodynamics) (Table 2). Two recent architectures—Joint-Embedding Predictive Architecture (JEPA) and Genie 3—exemplify this approach and offer a blueprint for its application to MD.

### JEPA: learning the essence of change

Yann LeCun, Chief AI Scientist at Meta, has proposed the JEPA as a path toward more general intelligence [7]. JEPA’s central idea is elegant and computationally efficient: rather than attempting to predict every detail of a system’s future state (e.g., every pixel in a video or every atomic coordinate in a protein), the model learns to predict the representation of that future state in an abstract latent space.

The JEPA principle operates as follows: the model is presented with a context (a short video, part of an image) and asked to predict the representation of a missing part (a future frame, a hidden region). By comparing its latent prediction with the actual representation of the missing part, the model learns the semantic relationships and intuitive physics connecting different parts of the system—without the prohibitive cost of pixel-level or atom-level generation.

This approach has already demonstrated viability in a complex biological domain. GeneJepa, a self-supervised foundation model, applies the JEPA architecture to learn a predictive world model of single-cell transcriptomes [8]. Rather than predicting expression levels of thousands of genes (a noisy, high-dimensional task), GeneJepa predicts latent representations of masked gene sets from visible context. In doing so, it learns functional relationships between genes and captures the underlying regulatory logic of cellular state. This powerful precedent demonstrates that JEPA principles can abstract the rules of a complex biological system.

### Genie 3: simulation of interactive and physically coherent worlds

In parallel, Google DeepMind has developed Genie 3, a general-purpose world model capable of generating interactive, photorealistic virtual worlds from simple text descriptions [9]. Genie 3 can simulate dynamic environments in real time, allowing a user to navigate and act within them while the model maintains physical and visual coherence over time. For example, if a user moves away from an object and then returns, the object remains in place, demonstrating spatial and temporal memory.

Genie 3’s most relevant capability for our proposal is “promptable world events.” A user can not only navigate but also introduce changes to the environment via text, such as “make it start raining.” The model responds by altering the simulation according to this new condition, demonstrating that it has learned a causal representation of how events function in the world.

The connection to protein dynamics is direct and potent. A molecular world model, trained on MD trajectories, could learn the “intuitive thermodynamics” of a protein. The “action” or “promptable event” would be the introduction of a ligand into the system. The model would not merely predict the final bound state but could “dream” or simulate the most probable conformational trajectory the protein follows to accommodate the ligand, revealing the induced fit mechanism in the process. Recent work by Ianeselli *et al* [10] has already explored the use of generative world models to compute protein folding pathways, demonstrating the viability of this approach.

### Transformative applications in oncology

We hypothesize that the adoption of world models to simulate protein dynamics is not merely an incremental advance; it represents a paradigm shift with the potential to unlock entirely new therapeutic strategies in oncology.

**Rational design of allosteric modulators.** Dynamic models might enable visualization of how perturbations at an allosteric site propagate through the protein structure to affect the active site. This could transform allosteric modulator discovery from a largely empirical screening process to a rational design exercise. We could design molecules not only to bind an allosteric cavity but to induce the precise conformational transition needed to inhibit (or activate) an oncoprotein.

**Drugging the “Undruggable.”** The universe of “undruggable” targets—including IDPs such as c-Myc, mutant p53, and transcription factors like STAT3—could potentially become more accessible. A world model could predict the transient “druggable conformational states” within an IDP’s dynamic ensemble. The goal would no longer be finding a drug for a structure, but designing a “molecular glue” or stabilizer capable of trapping and maintaining the protein in an inactive or degradation-prone conformation.

**Optimization of drug residence and binding kinetics.** A drug’s efficacy depends not only on its affinity (how strongly it binds) but also on its kinetics (how fast it binds and dissociates). Dynamic models could predict energy barriers for binding and dissociation, allowing medicinal chemists to optimize molecules for greater “residence” at the target, which often correlates with greater *in vivo* efficacy.

**Prediction of resistance mutations.** By simulating the effect of mutations on a protein’s conformational dynamics, we could anticipate which genetic changes might confer drug resistance. For example, a mutation might not directly affect the drug binding site but could alter the protein’s conformational landscape such that it can no longer adopt the conformation necessary for effective drug binding. This would enable proactive development of next-generation therapies.

### Challenges and perspective toward 2035

The path toward implementing molecular world models at proteome scale is ambitious and fraught with challenges. The principal obstacle is the scarcity of high-resolution experimental data on protein dynamics. While the Protein Data Bank contains hundreds of thousands of static structures, data on conformational trajectories—derived from techniques such as nuclear magnetic resonance (NMR) spectroscopy, time-resolved crystallography, or single-particle cryo-electron microscopy (cryo-EM) in different states—remain far more limited.

However, the synergy between AI and experimentation can create a virtuous cycle. World models, even when trained on limited data, can generate dynamic hypotheses to guide new experiments. In turn, data from these experiments will refine and improve the next generation of models. Integration of MD simulation data, despite its own timescale limitations, will also be crucial for pretraining these models, teaching them the fundamental principles of atomic physics before fine-tuning with experimental data.

Beyond data scarcity, several additional feasibility challenges merit explicit acknowledgment. First, the computational cost of training world models on high-dimensional molecular trajectories is enormous; even the most advanced graphics processing unit (GPU) clusters face practical limits when simulating systems containing thousands of atoms across biologically relevant timescales (microseconds to milliseconds). Current MD simulations typically cover nanoseconds to microseconds, leaving slower conformational transitions—those most relevant to induced fit and allostery—largely inaccessible. Enhanced sampling methods (e.g., metadynamics, replica exchange) partially address this, but transfer of *in silico* trajectories to real biological behavior remains unproven at scale. Second, model validation presents a fundamental challenge: unlike static structure prediction, where experimental X-ray or cryo-EM coordinates serve as a clear ground truth, dynamic trajectories cannot be directly compared against a single reference state. Experimental techniques such as single-molecule Förster resonance energy transfer (FRET), hydrogen–deuterium exchange mass spectrometry, and time-resolved cryo-EM provide partial constraints, but a comprehensive validation framework for conformational world models does not yet exist. Third, the speculative nature of several clinical applications proposed here—particularly the rational targeting of IDPs and the proactive prediction of resistance mutations—will require rigorous prospective validation before these approaches can influence therapeutic decision-making. We therefore present the roadmap below as a research agenda requiring sustained empirical scrutiny, not as a guaranteed timeline of clinical translation.

#### Roadmap toward 2035:

**Phase 1 (2025–2028): Proof of concept.** Development of the first world models at single-protein scale, trained on extensive MD data for well-characterized proteins (e.g., kinases, proteases). The objective will be to demonstrate the capacity to predict induced fit for known ligands and validate trajectories against existing experimental data.

**Phase 2 (2028–2032): Scaling and generalization.** Expansion of models to cover entire protein families. Development of architectures capable of generalizing across different protein topologies. Integration of heterogeneous experimental data (NMR, cryo-EM, etc.) into the training process. First successes in de novo design of allosteric modulators for well-validated targets.

**Phase 3 (2032–2035): Toward the dynamic proteome.** Development of proteome-scale world models capable of predicting the dynamics of any human protein. Integration with genomic models such as AlphaGenome to create a “digital twin” of the cell, linking genetic variation with changes in protein expression and dynamics. AI will not merely predict structures but simulate life at the molecular level.

### Conclusion

The AI revolution in biology has begun with prediction of the static form of life’s molecules. The coming decade will witness the transition from form to function, from structure to dynamics. World models, with their capacity to learn a system’s underlying rules and simulate its evolution, represent the key to unlocking this new frontier. By teaching AI to dream of protein motion, we will move from being mere observers of the molecular universe to becoming its architects. For oncology, this means a transition from target identification to active and precise modulation of their behavior, finally opening the door to treating cancers we today consider incurable.

### Conflicts of interest

The author declares no conflicts of interest relevant to the content of this article.

### Funding

This work received no specific grant from any funding agency in the public, commercial, or not-for-profit sectors.

### Disclosure of AI assistance

The author used Manus (manus.im) as a research assistant for information gathering, literature analysis, and manuscript drafting, and Claude (Anthropic) for syntax refinement, language polishing, and English translation. The intellectual content, scientific arguments, structure, and conclusions are entirely the author’s own.

## Figures and Tables

**Table 1.** Limitations of current AI models in the context of protein dynamics.

Model	Primary capability	Key dynamic limitation
AlphaFold 3	Biomolecular complex structure prediction	Generates static snapshot of bound state; does not model binding trajectory or induced fit
AlphaMissense	Missense variant pathogenicity prediction	Does not predict structural or dynamic changes due to mutation
AlphaProteo	De novo static protein binder design	Designs for static targets; does not design to modulate target dynamics
AlphaGenome	Regulatory variant effect prediction	Does not model how gene expression changes affect cellular proteome dynamics

**Table 2.** Comparison of static models versus the proposed world model approach.

Feature	Static models (e.g., AlphaFold 3)	World models (Proposed)
Primary objective	Predict a single low-energy 3D structure	Learn an internal model of system dynamics
Output	Atomic coordinates of a static state	Trajectories of conformational states over time
Analogy	A radiograph or high-resolution photograph	A movie or interactive simulation
Induced fit handling	Absent. Predicts final complex, not transition	Central. Simulates conformational response to ligand binding
Disordered proteins	Problematic. Tends to impose nonexistent structure	Potentially ideal. Models conformational ensemble and transitions
Computational basis	Prediction in output space (coordinates)	Prediction in latent space (abstract representations)
Drug applications	Static docking, binder design for rigid targets	Allosteric modulator design, IDP drugs, dynamic optimization
